# Drug-Coated Balloons and Bioresorbable Scaffolds in Spontaneous Coronary Artery Dissections

**DOI:** 10.3390/jcm14248751

**Published:** 2025-12-10

**Authors:** Marios Sagris, Marios G. Bantidos, Nikolaos Stalikas, Barbara Fyntanidou, Christos Kofos, Konstantinos Tsioufis, Efstratios Karagiannidis, Nikolaos Patsourakos

**Affiliations:** 1 1st Department of Cardiology, Hippokration General Hospital, National and Kapodistrian University of Athens, 11527 Athens, Greece; ktsioufis@gmail.com; 2 Department of Cardiology, “Tzaneio” General Hospital of Piraeus, 18542 Piraeus, Greece; drpats@yahoo.gr; 3 Second Department of Cardiology, General Hospital ‘Hippokration’, Aristotle University of Thessaloniki, Konstantinoupoleos 49, 54642 Thessaloniki, Greece; mbadidos@gmail.com (M.G.B.); stratoskarag@gmail.com (E.K.); 4 Cardiovascular Center, AZORG Ziekenhuis, Moorselbaan 164, 9300 Aalst, Belgium; nstalik@gmail.com; 5 Department of Emergency Medicine, AHEPA University Hospital, 54636 Thessaloniki, Greece; bfyntan@yahoo.com

**Keywords:** bioresorbable scaffold (BRS), drug-coated balloon (DCB), false lumen (FL), intramural hematoma (IMH), intravascular imaging, percutaneous coronary intervention (PCI), spontaneous coronary artery dissection (SCAD)

## Abstract

Spontaneous coronary artery dissection (SCAD) is an increasingly recognized cause of acute coronary syndromes in younger women without typical atherosclerotic risk factors. Its distinct pathophysiology and vessel fragility create unique challenges for revascularization. Conservative management is preferred when hemodynamics and coronary flow permit, but selected cases necessitate intervention, primarily percutaneous coronary intervention (PCI). Despite growing insights into SCAD pathomechanics—the “outside-in” and “inside-out” hypotheses—and the central role of intracoronary imaging (OCT/IVUS), optimal device strategies remain under-researched. The present review covers contemporary SCAD-PCI pitfalls and limitations, expanding to the mechanistic underpinnings and procedural applications of drug-coated balloons (DCB) and bioresorbable scaffolds (BRS) as “leave-nothing-behind” alternatives. Both approaches have advantages and drawbacks but are attractive in selected scenarios: DCB delivers antiproliferative therapy without permanent caging, and BRS provides temporary scaffolding (amenable to overlap when required) with the potential to restore biomechanics/vasomotion after resorption. Acknowledging that definitive evidence is lacking and current data are largely observational, the review finally sets future research priorities including head-to-head trials of different DCB types and evaluation of next-generation, thinner-strut, predictably resorbing BRS. The overarching question is whether—and how—these modalities can be integrated into standardized, imaging-guided interventional algorithms for SCAD.

## 1. Introduction

Spontaneous coronary artery dissection (SCAD) is an increasingly recognized cause of acute coronary syndromes (ACS), particularly in younger women without traditional atherosclerotic risk factors [1]. In the broader percutaneous coronary intervention (PCI) era, drug-eluting stents (DES) transformed coronary revascularization through advances in drug coatings and thinner struts [2]. However, compared with atherosclerotic disease, PCI in SCAD is uniquely challenging because of lesion anatomy and vessel fragility, with higher risks of technical failure and complications [3]. Accordingly, conservative management is the standard approach when hemodynamics and coronary flow permit.

Nevertheless, while SCAD can result in life-threatening complications, the optimal interventional strategy remains ill-defined. Contemporary practice largely adapts DES-based PCI for SCAD, accepting inherent trade-offs out of necessity. This review shifts the focus to minimally vessel-altering strategies—drug-coated balloons (DCB) and bioresorbable scaffolds (BRS)—which aim to restore flow while avoiding the long-term liabilities of permanent metallic stenting [3]. Emphasis is placed on the mechanistic rationale, procedural applications, and current evidence for DCB and BRS, alongside context-specific advantages and limitations. The aim is to lay the groundwork for targeted future research on these modalities and their implementation within validated, imaging-guided algorithms.

## 2. Pathophysiological Mechanisms

Two main pathophysiological hypotheses have been proposed to explain intramural hematoma (IMH) formation in SCAD. The “inside-out” hypothesis suggests that an initial intimal tear allows blood to enter the arterial wall from the lumen, leading to dissection and false lumen (FL) formation, similar to the mechanism of aortic dissection [4]. In contrast, the “outside-in” hypothesis posits that the IMH arises de novo within the tunica media, potentially from rupture of local microvasculature or the vasa vasorum. In this model, any observed intimal disruption or fenestration is considered a secondary event caused by FL pressurization by the IMH [5].

Mounting evidence strongly supports the outside-in mechanism as the dominant process in most SCAD cases. Intracoronary imaging studies reveal no identifiable flap or tear in approximately two-thirds of patients, indicating that the inciting event likely arises within the vessel wall [6,7]. On serial angiography alongside intracoronary imaging, IMH typically precedes any fenestration, and when a tear subsequently develops it often acts as a decompressive window—improving distal flow and relieving luminal compression [8]. Conversely, when no decompression occurs (non-fenestrated SCAD), features of FL pressurization appear with external elastic lamina expansion, larger FL area, and greater stenosis for a given lesion length [7]. These changes frequently regress during convalescence, suggesting that acute FL pressurization is transient.

Medial susceptibility is further supported by the high burden of arteriopathies/connective tissue disorders in SCAD; fibromuscular dysplasia is the most common (up to 50%) and is associated with tortuosity, cerebral aneurysms, and extracoronary dissections. Genetic variants affecting connective-tissue integrity and vascular structure further suggest a multifactorial basis for vessel-wall fragility [6].

Finally, it is noteworthy that although earlier work proposed that vasa vasorum rupture triggers IMH, recent optical coherence tomography (OCT) studies have not demonstrated consistently increased vasa vasorum density in acute SCAD compared with controls, implying a limited direct role in initiation [5,7]. Nonetheless, adventitial inflammation and neovascularization of the FL are often observed during healing, which may reflect a reparative response rather than a primary cause.

## 3. Angiographic Characteristics and Classification

Since SCAD most frequently manifests as ACS, particularly non-ST-elevation myocardial infarction (NSTEMI) (~70%), invasive coronary angiography interpreted alongside clinical context is often sufficient for diagnosis by experienced interventionalists [9]. SCAD lesions tend to affect the mid-to-distal coronary arteries, with the left anterior descending (LAD) artery most frequently involved (32–52% of cases), while left main (LM) and proximal segments are rarely affected (<8%). Multivessel involvement occurs in up to 10–15% of cases, though most SCAD events affect a single coronary segment [9].

The Yip-Saw angiographic classification outlines four SCAD patterns, reflecting the visual morphology of the dissected segment, and has been endorsed by the European Society of Cardiology (ESC) consensus panel [10]. The angiographic appearance of Type 1 SCAD is considered pathognomonic, characterized by a dual-lumen sign with contrast staining of the FL and a radiolucent intimal flap. It is seen in roughly 25–30% of SCAD cases and is thought to represent a later stage of dissection with a lower risk of acute progression [11]. Type 2 is the most common angiographic presentation (60–70%) and manifests as a long, smooth, diffuse narrowing (>20–30 mm), often tapering distally. These lesions do not respond to intracoronary nitroglycerin, helping differentiate them from vasospasm [12]. Type 3 lesions, which account for less than 10% of cases, appear as short (<20 mm), focal stenoses and are easily mistaken for atherosclerotic plaque, often necessitating OCT or intravascular ultrasound (IVUS) confirmation and may challenge even the most experienced operators. Finally, Type 4 describes total vessel occlusion and can be challenging to differentiate from thrombotic occlusion [12,13]. Observational data suggest that Types 2 and 3 are linked to higher rates of early reinfarction and unplanned Percutaneous Coronary Intervention (PCI), primarily driven by recurrent Myocardial Infarction (MI) and the need for revascularization [14].

## 4. Current Interventional Management: Indications and Challenges

The contemporary management of SCAD has shifted toward a conservative-first approach, increasing from approximately 35% before 2013 to nearly 90% of cases in recent years, driven by observational evidence of high spontaneous vessel healing rates and the technical challenges of revascularization [11,15]. Angiographic follow-up studies report spontaneous resolution of dissected segments in 70–97% of cases within weeks to months [16].

Nevertheless, a subset of SCAD patients requires urgent revascularization due to high-risk clinical or anatomical features. Intervention is typically reserved for patients with ongoing or recurrent ischemia, persistent chest pain or ST-segment elevation, hemodynamic or electrical instability, LM dissection, or occluded vessels with TIMI 0–1 flow [8,17]. Multivessel dissections, proximal LAD involvement, or a large myocardial territory at risk are also considered factors prompting invasive management when PCI is deemed technically feasible [17].

The therapeutic objective in SCAD differs from conventional PCI for atherosclerotic disease; the aim is not full vessel reconstruction but restoration of adequate coronary perfusion, typically defined as TIMI 3 flow, while minimizing procedural manipulation [11]. Despite this conservative philosophy, PCI outcomes in SCAD remain suboptimal. In the Canadian SCAD series, procedural success was achieved in only 64% of patients, and durable long-term results were maintained in just 30% [9,18]. Similarly, the Mayo Clinic reported a procedural success rate of 57%, and revascularization did not reduce the long-term risk of recurrent SCAD or the need for repeat intervention [19].

PCI in SCAD is associated with a wide spectrum of complications. Iatrogenic complications occur at a much higher rate in SCAD compared to standard PCI; iatrogenic coronary artery dissection occurs in 3–4% of SCAD interventions, compared with <0.2% in other PCI cases, while overall procedural complication rates have been reported as high as 22% [11,20]. IMH propagation and distal extension of the FL are particularly problematic, occurring in up to one-third of PCI cases, often necessitating additional unplanned stent implantation or endangering the patency of side branches [10,17]. The presence of IMH can impede adequate stent expansion, leading to late stent mal-apposition upon IMH resorption, while under-expansion further increases the risk of in-stent restenosis and thrombosis [17]. Another major technical challenge is inadvertent FL wiring—or even stenting—which can exacerbate the dissection, even as advanced FL-wiring techniques for flow restoration have been described [21]. Coronary artery perforation, though less frequent, remains a serious concern. Finally, late complications such as in-stent restenosis remain significant, with a substantial proportion of stented vessels requiring revascularization during follow-up [19].

## 5. Drug Coated Balloons

### 5.1. Rationale for Use in SCAD

Given that SCAD predominantly affects young women with otherwise healthy coronary arteries, there is a strong rationale for treatment strategies that mitigate aggressive vessel alteration. In recent years, the “leave-nothing-behind” concept has emerged as an intriguing research domain. The DCB have become the main representative, delivering a localized, temporary burst of antiproliferative drug to the vessel wall, thereby avoiding the permanent metallic scaffolding [22]. It has been demonstrated that even with modern DES platforms, the so-called “topological skeleton” of wall shear stress is altered, with potential downstream effects on endothelial signaling and long-term vascular health [23,24]. This concern is particularly relevant in SCAD, where changes in compliance over time may predispose to long-term complications [25,26]. Conversely, a DCB approach benefits from the absence of a stent layer, allowing the vessel wall to naturally remodel to its original configuration [22,27]. In this case, vessel stabilization of flow-limiting lesions is achieved by transiently sealing the intimal flap [28,29]. The importance of intracoronary imaging in SCAD is heightened when considering DCB, since their use will be beneficial in cases of confirmed intimal tear dissection as the inceptive event (“inside-out” mechanism). However, concerns have been raised related to potential impaired sealing with DCB in weaker wall integrity of SCAD [17]. In practice, during an “inside-out” SCAD, where intimal tear causes blood to enter the false lumen, intravascular imaging (OCT/IVUS) frequently reveals a double-lumen configuration or a focal entry point. When gentle ballooning or targeted fenestration decompresses the false lumen and restores true-lumen patency, DCB therapy can be preferable to stenting because it delivers antiproliferative drug without implanting a scaffold that may become malapposed as the false lumen collapses. In contrast, in “outside-in” SCAD which is characterized by diffuse IMH from vasa-vasorum bleeding without a clear entry tear, the long narrowing and pronounced compressive IMH often exhibit recoil after ballooning, making DCB less reliable and sometimes necessitating stenting if flow cannot be maintained. DCB should be avoided when persistent entry flow, proximal large dissections, or complex bifurcation anatomy mandate durable scaffolding [17].

In cases of iatrogenic coronary dissection after the use of DCB, the treatment strategy relies on not intervening when no flow limitation is observed. Studies report no excess thrombotic events at one year and angiographic healing on follow-up [30]. This contrasts with plain balloon angioplasty, where leaving a dissection has been associated with thrombosis, re-occlusion, and restenosis, prompting the adoption of stents as standard care [31]. These findings suggest that, if paclitaxel or sirolimus are effectively delivered to the vessel wall, DCB might potentially support natural healing. Consistent with these benefits, “uncaged” coronary arteries (due to no apposition of stent) have demonstrated improved late lumen gain in series of native CAD treated with DCB [32,33]. The evidence supporting DCB use in SCAD is derived almost entirely from small observational cohorts and case reports, which limits generalizability and introduces selection bias toward anatomically favorable or hemodynamically stable cases. Most series lack standardized imaging protocols, uniform criteria for DCB suitability, or long-term follow-up, making it difficult to compare outcomes across studies.

### 5.2. Evolution and Current Technologies

Until recently, paclitaxel had been standing as the drug of choice for most available DCB. Due to favorable lipophilic properties, paclitaxel achieves rapid membrane penetration and cellular uptake; then via binding to the microtubules, it inhibits their depolymerization, thus irreversibly blocking (“arresting”) cell division [34]. This mechanism explains the drug’s anti-inflammatory and anti-proliferative effects. Limus-based drugs (e.g., sirolimus), on the other hand, show strong safety and efficacy profiles but are less lipophilic, historically limiting tissue penetration and retention via DCB. However, technological advances have successfully overcome these challenges, with the encapsulation of the drug in a lipophilic nano-carrier leading to the development -limus DCB with remarkable efficacy in the treatment of both de novo coronary lesions and in-stent restenosis [34,35]. Biolimus A9, a sirolimus derivative with enhanced lipophilicity, is an additional emerging option while retaining rapamycin/mTOR-inhibitory activity [36]. Of note, rare coronary aneurysms have been reported after paclitaxel-coated balloons in de novo disease, and most DCB experience to date remains in in-stent restenosis [37]. Further studies are needed to define DCB use in SCAD specifically and to compare limus- vs. paclitaxel-based balloons in this specific setting.

## 6. Bioresorbable Scaffolds

### 6.1. Evolution and Current Technologies

The concept of BRS was first explored over three decades ago as a response to the limitations of permanent metallic stents, particularly their interference with vasomotor function, vessel remodeling, and long-term safety [38]. The idea was to transiently support the vessel during healing, then allow it to restore its native structure and function without residual foreign material, a strategy that eventually became known as “vascular reparative therapy”. Building on early polymeric platforms, second-generation systems achieved thinner struts, improved radial strength, deliverability, and rapid bioresorption [39,40]. Progress then culminated in a third-generation sirolimus-eluting magnesium scaffold (DREAMS 3G; Freesolve, Berlin, Germany), which has shown reassuring 3-year safety with favorable outcomes in terms of late lumen loss and target lesion failure.

### 6.2. Rationale for Use in SCAD

In the context of SCAD, a temporary scaffold would, similarly to DCB, allow recovery of vasomotor tone and minimize interference with endothelial integrity and mechanotransduction [23,24]. Moreover, the disease’s tendency for spontaneous resolution often makes extended radial support unnecessary, strengthening the case for a transient device [41].

Procedurally, most SCAD lesions occur in soft, non-calcified arteries, minimizing the need for aggressive lesion preparation. This vascular profile may help offset the deployment sensitivity of BRS, allowing for smoother delivery and reducing the risk of procedural trauma. In addition, since the dissected segments are typically long and tapering, BRS can address the matter by delivering full-length coverage via overlapping [42]. Another important consideration is the potential for shorter dual antiplatelet therapy (DAPT) duration, a meaningful advantage for younger patients or those with bleeding risk or future pregnancy considerations [25]. Finally, the absence of permanent intravascular hardware/fixed metallic struts preserves the ability to perform future PCI or surgical bypass [43].

### 6.3. Available Evidence

Large, randomized trials assessing the use of BRS in SCAD are currently lacking, with most of the available evidence stemming from observational case series, registries and individual case reports. Possibly the largest cohort of SCAD patients treated with BRS reported to date comes from Macaya et al., who described outcomes in 22 high-risk individuals treated with the Absorb BRS, with a median follow-up of 3.5 years [44]. The cohort was representative of the SCAD population (predominantly young women with minimal atherosclerosis and mid-to-distal segment involvement). Importantly, there were no deaths, myocardial infarctions, or scaffold thrombosis events during follow-up. Only one device-related target lesion revascularization was reported, due to scaffold shrinkage, while a second patient underwent revascularization for a residual flap beyond the scaffolded area. Consistent findings were reported in a slightly smaller multicenter series by Ielasi et al., who analyzed 18 SCAD cases from the “Registro Absorb Italiano” (RAI) Registry managed with Absorb BRS implantation [45]. All patients presented with ACS, and successful scaffold deployment was achieved in every case, often requiring multiple overlapping devices (61%). TIMI 3 flow was restored in all cases, and no adverse clinical events occurred during hospitalization or at a median follow-up of 18 months. This study also emphasized the importance of imaging support, with intracoronary imaging used in 50% of patients to guide device sizing and placement. Notably, these outcomes were achieved despite lesion complexity, including frequent mid-LAD involvement (56%) and long dissection segments.

Real-world experiences reinforce the feasibility of BRS use in SCAD. Multiple case reports have documented successful BRS use in a variety of anatomically and clinically complex scenarios, including left main, mid-LAD, and long right coronary artery (RCA) dissections. The management of these cases often involved sequential or overlapping scaffold implantation and intravascular guidance by OCT or IVUS with follow-up durations ranging from 6 to 40 months [41,46,47,48]. Published experience with BRS in SCAD is limited, consisting predominantly of highly selected cases treated in expert centers. Sample sizes are small, and procedural success is often influenced by operator experience and imaging-guided case selection, which may not reflect routine practice. We need more comparative data and longer follow-up periods after scaffold apposition to establish the BRS use in everyday practice.

### 6.4. Procedural Considerations and Imaging Guidance

The technical deployment of BRS in SCAD requires heightened procedural precision due to both the device’s mechanical properties and the fragility of the affected vessel. Unlike metallic DES, BRS platforms are more susceptible to underexpansion, malapposition, or strut fracture if not properly sized and delivered [49]. As such, optimal lesion preparation and accurate scaffold sizing are critical. While aggressive pre-dilatation is usually unnecessary in SCAD due to the absence of calcification, accurate assessment of dissection length and vessel tapering is essential, particularly when overlapping scaffolds are anticipated. Vessel sizing must be approached conservatively, as SCAD-affected segments often appear smaller due to IMH compression. The operators should size to the estimated true lumen using intravascular imaging rather than angiography alone. Long dissections may necessitate overlapping scaffolds, but this should be minimized given the increased risk of scaffold thrombosis and loss of physiological taper. In vessels with marked tapering, careful stepwise upsizing or focal post-dilation may help match the natural diameter gradient while avoiding oversizing-induced propagation of the dissection. Intravascular imaging is invaluable for delineating the proximal and distal edges of flow-limiting dissections and for distinguishing focal IMH from atherosclerotic plaque rupture in ambiguous cases. IMH fenestration can be achieved with low-pressure ballooning or cutting-balloon micropuncture decompressing the hematoma before scaffold deployment, improving true-lumen expansion and reducing the need for excessive post-dilation. Together, these considerations help optimize BRS implantation in anatomically complex SCAD lesions while mitigating the risks inherent to treating fragile, dissected vessels [49].

In cases of well-defined entry and exit tears, BRS may provide temporary scaffolding to seal the lesion while minimizing long-term malapposition or thrombosis risk. Several reports support this approach with favorable angiographic and clinical outcomes, though late malapposition has occasionally been observed on follow up imaging [46,47]. When obstructive IMH are present, lesion preparation with a cutting balloon to fenestrate the intima and decompress the IMH is reasonable. If flow does not improve, BRS implantation with adequately long proximal and distal landing zones may be considered. Notably, BRS should be avoided in vessels <2.5 mm due to the elevated thrombosis risk observed in previous trials, making distal dissections unsuitable for this approach [25,28]. Finally, post-procedural imaging may be valuable in assessing immediate scaffold integration and subsequent vessel healing, although its routine use in SCAD cases treated with BRS remains investigational.

## 7. Future Research Directions

Despite growing recognition of SCAD as a notable cause of ACS, optimal interventional management remains insufficiently characterized. First and foremost, there is a need for detailed patient selection. A priority is the development and external validation of risk stratification models that integrate clinical presentation, angiographic phenotype, and hemodynamic indices, while explicitly incorporating sex-specific and pregnancy-related variables [17]. In tandem, refinement of intravascular imaging—particularly OCT and IVUS—should enable precise delineation of the precipitating mechanism, the extent of FL and IMH, and suitable landing zones, thereby translating anatomy into actionable procedural plans [50].

Transformative advances are most likely to emerge from SCAD-tailored device innovation. Engineering priorities should include sizing algorithms calibrated for small and fragile vessels, optimized drug formulations and coatings that balance tissue uptake, and scaffold architectures with lower strut thickness, and predictable, homogenous resorption kinetics. At the same time, it is important to refine procedural techniques—such as wire selection, IMH decompression strategies, and judicious hybrid approaches—in which DCB therapy is complemented by focal scaffolding (e.g., to secure flow or seal entry points) [51,52,53]. Operationally, three programmatic steps follow from this agenda: (a) institution of OCT/IVUS-guided protocols that define landing zones and the longitudinal burden of IMH before device selection; (b) systematic evaluation of DCB lesion-preparation techniques—encompassing balloon sizing and controlled fenestration—to achieve expansion while minimizing IMH propagation; and (c) prospective, controlled assessment of next-generation BRS in dedicated SCAD cohorts and registries, with prespecified imaging and clinical endpoints capturing both early safety and late vascular healing.

Nevertheless, although early reports of DCB and BRS use in SCAD are encouraging, the evidence base remains limited—largely case reports, small case series, and extrapolation from non-SCAD-PCI studies. Accordingly, these modalities are selectively reserved for experienced operators, pending adequately powered studies to establish short- and long-term safety and efficacy. For pregnancy-associated and peripartum SCAD, dedicated pathways are essential—minimizing radiation, optimizing timing of intervention, and integrating maternal–fetal medicine within heart-team decision-making [54]. Taken together, these directions underscore the need for diagnostic precision, rigorous patient selection, and device choice, with clear emphasis on tailoring interventional strategies to the unique pathomechanics of SCAD.

A concise comparison of DES, DCB, and BRS in SCAD-PCI is provided in Table 1.

## 8. Conclusions

When PCI is warranted, early data suggest that stent-less strategies—using DCB or BRS—may help restore flow while preserving endothelial integrity and vasomotor reactivity. Ongoing studies are expected to clarify patient selection, standardize imaging-guided techniques, and support the development of a coherent, evidence-based treatment algorithm for complex SCAD presentations.

## Figures and Tables

**Table 1:** DES vs. DCB vs. BRS in SCAD-PCI: key comparative features.

Feature	DES	DCB	BRS
**Implant status**	Metallic scaffold	No implant	Temporary scaffold
**Vasomotion restoration**	Impaired	Preserved	Preserved
**Acute flow restoration**	Flow restoration	Restore flow Considered in “inside-out” mechanism	Long, flow-limiting dissections with defined entry/exit
**Resistance to recoil**	High	Limited	Moderate early wanes as scaffold resorbs
**Iatrogenic risk**	Higher overall complication and extension rates vs. routine PCI; common IMH propagation	Avoids permanent metal but still requires gentle wiring/imaging to prevent propagation	Deployment-sensitive; careful sizing/landing
**Early/Late thrombosis and DAPT**	Low with new-generation DES	Very low Shorter DAPT	Early hazard Shorter DAPT after healing
**Suitability in tortuous vessels**	Cage distorts geometry; Frequent tortuosity in SCAD	Conforms to natural curvature	Challenging delivery in acute phase
**Suitability for long segment**	Potential “full metal jacket”	Uncertain sealing	Overlap possible
**Intravascular imaging**	Confirm mechanism Sizing	Confirm mechanism and true tear	Central for sizing/landing/overlap
**Future PCI or CABG**	Crossing/landing limitations through stented segments	Fully preserved	Preserved after resorption
**Current evidence in SCAD**	Higher complication rates vs. routine PCI	Early series/experience suggest feasibility Limited Evidence	Good mid-term outcomes Case series/registries Limited Evidence
**Future directions**	-	Head-to-head limus vs. paclitaxel in SCAD Standardized lesion prep./IMH decompression protocols	Next-gen BRS evaluation (thinner struts, predictable resorption) OCT/IVUS-guided algorithms

A practical summary of differences across DES, DCB, and BRS in SCAD. Emphasis is placed on mechanistic and procedural aspects: BRS: bioresorbable scaffold; CABG: coronary artery bypass grafting; DAPT: dual antiplatelet therapy; DCB: drug-coated balloon; DES: drug-eluting stent; IMH: intramural hematoma; IVUS: intravascular ultrasound; OCT: optical coherence tomography; PCI: percutaneous coronary intervention; SCAD: spontaneous coronary artery dissection.

## Data Availability

The data generated in this research will be shared on reasonable request to the corresponding author.

## Abbreviations

ACS: acute coronary syndromes; BRS: bioresorbable scaffold; CAD: coronary artery disease; DAPT: dual antiplatelet therapy; DCB: drug-coated balloon; DES: drug-eluting stent; DREAMS 3G: sirolimus-eluting magnesium scaffold; ESC: European Society of Cardiology; FL: false lumen; IMH: intramural hematoma; IVUS: intravascular ultrasound; LAD: left anterior descending; LM: left main; MI: myocardial infarction; mTOR: mechanistic target of rapamycin; NSTEMI: non-ST-elevation myocardial infarction; OCT: optical coherence tomography; PCI: percutaneous coronary intervention; RAI: Registro Absorb Italiano; RCA: right coronary artery; SCAD: spontaneous coronary artery dissection; TIMI: Thrombolysis In Myocardial Infarction.

## References

[1] Bcharah H., Bcharah G., Nabi H.A., Dreher L., Ibrahim R., Abdelnabi M., Pham H.N., Kanaan C., Kumar S., Baudhuin L.M. (2025). Extracoronary Arterial Pathologies in Patients With Spontaneous Coronary Artery Dissection. Am. J. Cardiol..

[2] Capodanno D. (2016). Very Late Outcomes of Drug-Eluting Stents: The “catch-down” Phenomenon. Eur. Heart J..

[3] Narducci M.L., Flore F., Simonini C., Carmina V., Montone R.A., Pinnacchio G., Bencardino G., Perna F., Animati F.M., Tremamunno S. (2025). High Incidence of Adverse Events in Spontaneous Coronary Artery Dissection Patients during Mid-Term Follow-up: A Persistent Challenge Ahead. Minerva Cardiol. Angiol..

[4] Waterbury T.M., Tweet M.S., Hayes S.N., Eleid M.F., Bell M.R., Lerman A., Singh M., Best P.J.M., Lewis B.R., Rihal C.S. (2018). Early Natural History of Spontaneous Coronary Artery Dissection. Circ. Cardiovasc. Interv..

[5] Hayes S.N., Tweet M.S., Adlam D., Kim E.S.H., Gulati R., Price J.E., Rose C.H. (2020). Spontaneous Coronary Artery Dissection: JACC State-of-the-Art Review. J. Am. Coll. Cardiol..

[6] Crousillat D., Sarma A., Wood M., Naderi S., Leon K., Gibson C.M., Aday A., Grodzinsky A., Izard K., Kovacic J.C. (2024). Spontaneous Coronary Artery Dissection: Current Knowledge, Research Gaps, and Innovative Research Initiatives: JACC Advances Expert Panel. JACC Adv..

[7] Jackson R., Al-Hussaini A., Joseph S., van Soest G., Wood A., Macaya F., Gonzalo N., Cade J., Caixeta A., Hlinomaz O. (2019). Spontaneous Coronary Artery Dissection: Pathophysiological Insights From Optical Coherence Tomography. JACC Cardiovasc. Imaging.

[8] Offen S., Yang C., Saw J. (2024). Spontaneous Coronary Artery Dissection (SCAD): A Contemporary Review. Clin. Cardiol..

[9] Saw J., Starovoytov A., Humphries K., Sheth T., So D., Minhas K., Brass N., Lavoie A., Bishop H., Lavi S. (2019). Canadian Spontaneous Coronary Artery Dissection Cohort Study: In-Hospital and 30-Day Outcomes. Eur. Heart J..

[10] Adlam D., Alfonso F., Maas A., Vrints C., Writing Committee (2018). European Society of Cardiology, Acute Cardiovascular Care Association, SCAD Study Group: A Position Paper on Spontaneous Coronary Artery Dissection. Eur. Heart J..

[11] Brunton N., Best P.J.M., Skelding K.A., Cendrowski E.E. (2024). Spontaneous Coronary Artery Dissection (SCAD) from an Interventionalist Perspective. Curr. Cardiol. Rep..

[12] Saw J. (2014). Coronary Angiogram Classification of Spontaneous Coronary Artery Dissection. Catheter. Cardiovasc. Interv..

[13] Eleid M.F., Guddeti R.R., Tweet M.S., Lerman A., Singh M., Best P.J., Vrtiska T.J., Prasad M., Rihal C.S., Hayes S.N. (2014). Coronary Artery Tortuosity in Spontaneous Coronary Artery Dissection: Angiographic Characteristics and Clinical Implications. Circ. Cardiovasc. Interv..

[14] Mori R., Macaya F., Giacobbe F., Salinas P., Pavani M., Boi A., Bettari L., Rolfo C., Porto I., Gonzalo N. (2021). Clinical Outcomes by Angiographic Type of Spontaneous Coronary Artery Dissection. EuroIntervention.

[15] Feldbaum E., Thompson E.W., Cook T.S., Sanghavi M., Wilensky R.L., Fiorilli P.N., Lewey J. (2023). Management of Spontaneous Coronary Artery Dissection: Trends over Time. Vasc. Med..

[16] Bollati M., Ercolano V., Mazzarotto P. (2024). Spontaneous Coronary Dissection Review: A Complex Picture. Rev. Cardiovasc. Med..

[17] Morena A., Giacobbe F., De Filippo O., Angelini F., Bruno F., Siliano S., Giannino G., Dusi V., Bianco M., Biolé C. (2024). Advances in the Management of Spontaneous Coronary Artery Dissection (SCAD): A Comprehensive Review. Rev. Cardiovasc. Med..

[18] Saw J., Starovoytov A., Aymong E., Inohara T., Alfadhel M., McAlister C., Samuel R., Grewal T., Parolis J.A., Sheth T. (2022). Canadian Spontaneous Coronary Artery Dissection Cohort Study: 3-Year Outcomes. J. Am. Coll. Cardiol..

[19] Tweet M.S., Eleid M.F., Best P.J.M., Lennon R.J., Lerman A., Rihal C.S., Holmes D.R., Hayes S.N., Gulati R. (2014). Spontaneous Coronary Artery Dissection: Revascularization versus Conservative Therapy. Circ. Cardiovasc. Interv..

[20] Prakash R., Starovoytov A., Heydari M., Mancini G.B.J., Saw J. (2016). Catheter-Induced Iatrogenic Coronary Artery Dissection in Patients With Spontaneous Coronary Artery Dissection. JACC Cardiovasc. Interv..

[21] Bonnet M., Toleva O., Ybarra L.F., Rinfret S. (2019). Successful Treatment of Spontaneous Coronary Artery Dissection With Subintimal Tracking and Re-Entry Technique. JACC Case Rep..

[22] Patel A.H., Abdelnour M.W., Frangieh A.H. (2025). Drug-Coated Balloons for Coronary Interventions: A Focused Review. Methodist. Debakey Cardiovasc. J..

[23] Chiastra C., Mazzi V., Lodi Rizzini M., Calò K., Corti A., Acquasanta A., De Nisco G., Belliggiano D., Cerrato E., Gallo D. (2022). Coronary Artery Stenting Affects Wall Shear Stress Topological Skeleton. J. Biomech. Eng..

[24] Traub O., Berk B.C. (1998). Laminar Shear Stress. Arterioscler. Thromb. Vasc. Biol..

[25] Karim Galougahi K., Ben-Yehuda O., Maehara A., Mintz G.S., Stone G.W., Ali Z.A. (2016). “The Scaffolding Must Be Removed Once the House Is Built”-Spontaneous Coronary Artery Dissection and the Potential of Bioresorbable Scaffolds. J. Thorac. Dis..

[26] Saw J., Mancini G.B.J., Humphries K.H. (2016). Contemporary Review on Spontaneous Coronary Artery Dissection. J. Am. Coll. Cardiol..

[27] O’Callaghan D., Rai H., Giacoppo D., Coughlan J.J., Durand R., Paradies V., Colleran R., Blake G.J., Alfonso F., Byrne R.A. (2025). Drug Coated Balloons Versus Drug-Eluting Stents in Patients With De Novo Coronary Artery Disease. Catheter. Cardiovasc. Interv..

[28] Wu S., Liu W., Zhao Y., Shi D., Liu R., Wang J., Yu Y., Jia S., Sun Y., Zhou Y. (2016). Revascularization Treatment for Spontaneous Coronary Artery Dissection: A Reconsideration of Drug-Coated Balloons and Bioresorbable Vascular Scaffold. Int. J. Cardiol..

[29] Buccheri D., Piraino D., Cortese B. (2016). Spontaneous Coronary Artery Dissection: A Hint into Its Diagnosis and Therapy. Int. J. Cardiol..

[30] Cortese B., Silva Orrego P., Agostoni P., Buccheri D., Piraino D., Andolina G., Seregni R.G. (2015). Effect of Drug-Coated Balloons in Native Coronary Artery Disease Left With a Dissection. JACC Cardiovasc. Interv..

[31] Cowley M.J., Dorros G., Kelsey S.F., Van Raden M., Detre K.M. (1984). Acute Coronary Events Associated with Percutaneous Transluminal Coronary Angioplasty. Am. J. Cardiol..

[32] Kleber F.X., Schulz A., Waliszewski M., Hauschild T., Böhm M., Dietz U., Cremers B., Scheller B., Clever Y.P. (2015). Local Paclitaxel Induces Late Lumen Enlargement in Coronary Arteries after Balloon Angioplasty. Clin. Res. Cardiol..

[33] Olsen P.R., Andersen H.H., Hebjørn M., Pedersen V.M., Hansen L.K. (1983). The Prophylaxis of Metronidazole in Colorectal Surgery. Dan. Med. Bull..

[34] Pasquier E., Carré M., Pourroy B., Camoin L., Rebaï O., Briand C., Braguer D. (2004). Antiangiogenic Activity of Paclitaxel Is Associated with Its Cytostatic Effect, Mediated by the Initiation but Not Completion of a Mitochondrial Apoptotic Signaling Pathway. Mol. Cancer Ther..

[35] Cortese B., di Palma G., Latini R.A., Elwany M., Orrego P.S., Seregni R.G. (2017). Immediate and Short-Term Performance of a Novel Sirolimus-Coated Balloon during Complex Percutaneous Coronary Interventions. The FAtebenefratelli SIrolimus COated-Balloon (FASICO) Registry. Cardiovasc. Revascularization Med..

[36] Traynor B.P., Fitzgerald S., Alfonso F., O’Kane P., Sabaté M., Tölg R., Trevelyan J., Hahn J.-Y., Mylotte D., Wöhrle J. (2023). Design and Rationale of a Prospective, Randomized, Non-Inferiority Trial to Determine the Safety and Efficacy of the Biolimus A9^TM^ Drug Coated Balloon for the Treatment of in-Stent Restenosis: First-in-Man Trial (REFORM). Cardiovasc. Revasc. Med..

[37] Saboe A., Upadhya C., Finn A., Basavarajaiah S. (2025). Coronary Artery Aneurysm Post Drug-Coated Balloon Angioplasty. JACC Case Rep..

[38] Serruys P.W., Ormiston J.A., Onuma Y., Regar E., Gonzalo N., Garcia-Garcia H.M., Nieman K., Bruining N., Dorange C., Miquel-Hébert K. (2009). A Bioabsorbable Everolimus-Eluting Coronary Stent System (ABSORB): 2-Year Outcomes and Results from Multiple Imaging Methods. Lancet.

[39] Haude M., Ince H., Abizaid A., Toelg R., Lemos P.A., von Birgelen C., Christiansen E.H., Wijns W., Neumann F.-J., Kaiser C. (2016). Sustained Safety and Performance of the Second-Generation Drug-Eluting Absorbable Metal Scaffold in Patients with de Novo Coronary Lesions: 12-Month Clinical Results and Angiographic Findings of the BIOSOLVE-II First-in-Man Trial. Eur. Heart J..

[40] Ozaki Y., Garcia-Garcia H.M., Shlofmitz E., Hideo-Kajita A., Waksman R. (2020). Second-Generation Drug-Eluting Resorbable Magnesium Scaffold: Review of the Clinical Evidence. Cardiovasc. Revascularization Med..

[41] Cockburn J., Yan W., Bhindi R., Hansen P. (2014). Spontaneous Coronary Artery Dissection Treated with Bioresorbable Vascular Scaffolds Guided by Optical Coherence Tomography. Can. J. Cardiol..

[42] Panoulas V.F., Ielasi A. (2016). Bioresorbable Scaffolds and Drug-Eluting Balloons for the Management of Spontaneous Coronary Artery Dissections. J. Thorac. Dis..

[43] Camacho Freire S.J., Gómez Menchero A.E., Roa Garrido J., León Jiménez J., Cardenal Piris R., Díaz Fernández J.F. (2017). Bioresorbable Scaffolds in Spontaneous Coronary Artery Dissection: Long-Term Follow-Up in 4 Patients. Tex. Heart Inst. J..

[44] Macaya F., Salinas P., Gonzalo N., Camacho-Freire S.J., Jackson R., Massot M., Ortas-Nadal M.R., Sánchez-Recalde Á., Díaz-Fernández J.F., Moreu J. (2019). Long-Term Follow-up of Spontaneous Coronary Artery Dissection Treated with Bioresorbable Scaffolds. EuroIntervention.

[45] Ielasi A., Cortese B., Tarantini G., Loi B., Mazzarotto P., Gabrielli G., Tespili M., Rovera C., Corrado D., Steffenino G. (2016). Sealing Spontaneous Coronary Artery Dissection with Bioresorbable Vascular Scaffold Implantation: Data from the Prospective “Registro Absorb Italiano” (RAI Registry). Int. J. Cardiol..

[46] Watt J., Egred M., Khurana A., Bagnall A.J., Zaman A.G. (2016). 1-Year Follow-Up Optical Frequency Domain Imaging of Multiple Bioresorbable Vascular Scaffolds for the Treatment of Spontaneous Coronary Artery Dissection. JACC Cardiovasc. Interv..

[47] Gonzalo N., Macaya F., Gómez de Diego J.J., Escaned J. (2017). Long-Term Outcome of a Spontaneous Coronary Artery Dissection Treated with a Bioresorbable Scaffold. EuroIntervention.

[48] Sengottuvelu G., Rajendran R., Dattagupta A. (2015). Optical Coherence Tomographic Image of Dynamic Left Main Coronary Artery Compression Caused by Intramural Haematoma Due to Spontaneous Coronary Artery Dissection-Degloved Artery Managed with Bioresorbable Vascular Scaffold. EuroIntervention.

[49] Cortese B., Buccheri D. (2016). Acutely Malapposed Bioresorbable Vascular Scaffold during Primary Angioplasty for Spontaneous Coronary Artery Dissection: Is a No-Metal Strategy Better?. Int. J. Cardiol..

[50] Berisha L., Camaj A., Sharma S.K. (2025). Current and Future Role of Drug-Coated Balloons in the Treatment of Coronary Artery Disease. Future Cardiol..

[51] Xu K., Fu G., Wang X., Song L., Tao L., Li L., Hou Y., Su X., Fang Q., Chen L. (2025). 5-Year Outcomes With a Novel Coronary Sirolimus-Eluting Bioresorbable Scaffold: The NeoVas Randomized Trial. JACC Cardiovasc. Interv..

[52] Yu Y., Jiao Y., Wang L., Zhang M., Yin C. (2025). Research Status and Trends of Drug-Coated Balloons in Coronary Artery Disease: A Bibliometric Analysis. Front. Med..

[53] Ng K.Y., Muhammad N., Saleh M.S., Mohd Noor S.N.F., Muhammad N.A., Muduli K., Bupesh Raja V.K., Chong K.V. (2025). The Potential of Stent Cell Geometry to Affect Endothelialisation Performance: A Review of Existing Research and Future Perspective. Biomed. Mater..

[54] Thordsen S., Briller J., Cruz M.O. (2025). Cardiac Considerations in Pregnancy: A Spotlight on Peripartum Cardiomyopathy and Pregnancy-Associated Spontaneous Coronary Dissection. Obstet. Gynecol. Clin. N. Am..

